# Quick and Clean Cloning: A Ligation-Independent Cloning Strategy for Selective Cloning of Specific PCR Products from Non-Specific Mixes

**DOI:** 10.1371/journal.pone.0020556

**Published:** 2011-06-02

**Authors:** Frank Thieme, Carola Engler, Romy Kandzia, Sylvestre Marillonnet

**Affiliations:** Icon Genetics GmbH, Halle, Germany; Ohio State University, United States of America

## Abstract

We have developed an efficient strategy for cloning of PCR products that contain an unknown region flanked by a known sequence. As with ligation-independent cloning, the strategy is based on homology between sequences present in both the vector and the insert. However, in contrast to ligation-independent cloning, the cloning vector has homology with only one of the two primers used for amplification of the insert. The other side of the linearized cloning vector has homology with a sequence present in the insert, but nested and non-overlapping with the gene-specific primer used for amplification. Since only specific products contain this sequence, but none of the non-specific products, only specific products can be cloned. Cloning is performed using a one-step reaction that only requires incubation for 10 minutes at room temperature in the presence of T4 DNA polymerase to generate single-stranded extensions at the ends of the vector and insert. The reaction mix is then directly transformed into *E. coli* where the annealed vector-insert complex is repaired and ligated. We have tested this method, which we call quick and clean cloning (QC cloning), for cloning of the variable regions of immunoglobulins expressed in non-Hodgkin lymphoma tumor samples. This method can also be applied to identify the flanking sequence of DNA elements such as T-DNA or transposon insertions, or be used for cloning of any PCR product with high specificity.

## Introduction

One problem in molecular biology consists of identifying unknown sequences that flank a region of known sequence. Examples of applications where such problem is encountered include the determination of flanking sequences of stably integrated transgenes (e.g. T-DNA), the sequence flanking a transposon insertion, or the sequences of the variable regions of an immunoglobulin. In all cases, PCR cannot be used directly to amplify a fragment containing the known and unknown sequence since only the sequence at one end of the fragment to amplify is known. However, over the years, many protocols have been developed to bypass this problem and allow the identification of unknown flanking sequences. Such protocols cover a wide range of approaches, including inverse PCR [1], Tail PCR [2] and adaptor PCR [3], [4], [5] for DNA targets, and 5′ RACE for RNA targets [6], [7]. Basically, most of these protocols rely on attaching an adapter sequence to the end of the unknown sequence and using PCR for amplification of a fragment containing both known and unknown flanking sequences using a first primer binding to the adaptor sequence and a second primer binding to the known sequence. Since for all of these protocols the adaptor sequence is not exclusively attached to the desired sequence, many non-specific products are also amplified in a first PCR. Therefore, one or two additional PCR amplifications performed using nested primers binding in the known region are usually necessary to increase the ratio of specific to non-specific products. Identification of the unknown sequence can then be done simply by sequencing the amplified product with a nested gene-specific primer. However, if several specific products are expected to be amplified in the same reaction (for example a DNA sample may contain several transgenes and therefore several different flanking sequences, or an RNA sample extracted from a B-cell population will contain a large number of different immunoglobulin variable regions), direct sequencing will not be useful. Rather, the amplified products have to be cloned, and recombinant plasmids individually sequenced.

There are many approaches available for cloning of PCR products. Standard techniques that rely on digestion of insert and vector with restriction enzymes are not well suited for cloning fragments containing unknown sequences since presence of restriction sites in the unknown region may prevent cloning of such sequences. A number of techniques that do not require digestion of the inserts with restriction enzymes have been developed, including blunt-end cloning, cloning with topoisomerase, recombinase-based cloning and ligation-independent cloning (LIC) [8], [9]. Among these techniques, LIC presents many advantages. LIC is simple to perform and can be done using common reagents found in any molecular biology laboratory, and therefore does not require the purchase of a kit, but is nevertheless very efficient. The principle of the LIC strategy is based on regions of homology present in the primers used for amplification of the PCR product and the ends of a linearized cloning vector. Vector and insert are treated with an exonuclease such as T4 DNA polymerase or exonuclease III [8], [10], leading to formation of complementary single-stranded DNA overhangs that are able to anneal with each other. Annealed vector-insert complexes can be transformed directly in *E. coli* cells without ligation [8], [11].

One limitation for cloning of PCR products containing unknown flanking sequences is that a substantial fraction of the products can be non-specific. As described above, one source of non-specific products consists of sequences amplified with the adaptor primer only. Other non-specific products can be produced by non-specific annealing of one or both primers during amplification. Finally primer-dimers are a source of non-specific products that can occur during any PCR amplification. Due to the requirement for specific sequences on both sides of the insert, ligation-independent cloning should not lead to cloning of the non-specific products that result from amplification from a single primer. However, all other non-specific products can theoretically still be cloned.

To avoid this drawback, we have developed a ligation-independent cloning strategy, called quick and clean cloning, that allows cloning of specific products only. Here, we have tested this method for cloning of immunoglobulin variable regions amplified from cDNAs of non-Hodgkin lymphoma biopsy samples.

## Results

### Principle of the QC cloning strategy

**Figure 1:**
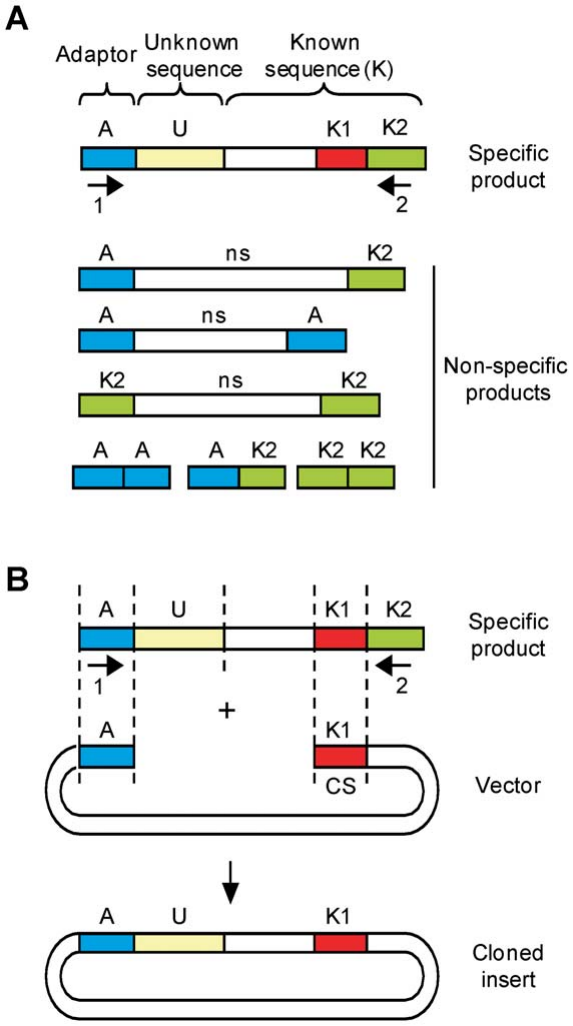
Principle of the QC cloning strategy. (**A**) PCR products amplified to identify unknown sequences flanking a region of known sequence typically consist of an adaptor sequence (A) attached to the end of the unknown sequence (U) followed by a region of known sequence (K). The PCR product is amplified with two primers (1 and 2) that are homologous to the adaptor sequence and to part of the known sequence (region K2). Non-specific products (ns) and primer dimers can also be obtained during PCR amplification. (**B**) The fragment is cloned by homology with a linearized vector that is homologous to the adaptor sequence at one end and to a sequence from the known region (K1, called the CS in the cloning vector) at the other end. Since sequence K1 does not overlap with sequence K2, non-specific products and primer dimers cannot be cloned.

Many protocols have been developed to amplify unknown sequences that flank known sequences using PCR [1], [2], [3], [4], [5], [6], [7]. The PCR products obtained typically contain adaptor sequences (A, **Fig. 1A**) attached to the end of a fragment of unknown sequence (U), followed by a fragment of known sequence (K). The PCR products are obtained by amplification with a first primer binding to the adaptor sequence (primer 1) and a second primer (primer 2) that anneals to part of the known sequence (sequence K2, arbitrarily defined as the sequence of the known region homologous to primer 2). In addition to specific products, several types of non-specific products (ns) can also be amplified (**Fig. 1A**).

The principle of the quick and clean (QC) cloning strategy is based on homology between sequences present in both the vector and the PCR product. However, in contrast to other ligation-independent strategies, the cloning vector has homology with only one of the two primers used for amplification, the primer designed to bind to the adaptor sequence (primer 1, **Fig. 1B**). The vector has no homology at all with the second primer (primer 2). Instead, the vector has homology with a sequence from the known region, sequence K1, located downstream of K2 (sequence K1 is defined as the sequence present in the vector, and expected to be present in the insert). The sequence fragment of the cloning vector corresponding to the K1 sequence is also referred to as the ‘catching sequence’ (CS). The advantage of this design is that only specific PCR products can be cloned, since only these contain region K1. Cloning by homology is performed as for ligation-independent cloning: a 3′ to 5′ exonuclease such as T4 DNA polymerase is used to generate complementary single-stranded ends for the PCR product and the cloning vector. For QC cloning, a longer single-stranded overhang needs to be generated than for LIC since both regions, K1 and K2, in the PCR product need to be made single-stranded.

### Quantification of T4 DNA polymerase exonuclease activity

**Figure 2:**
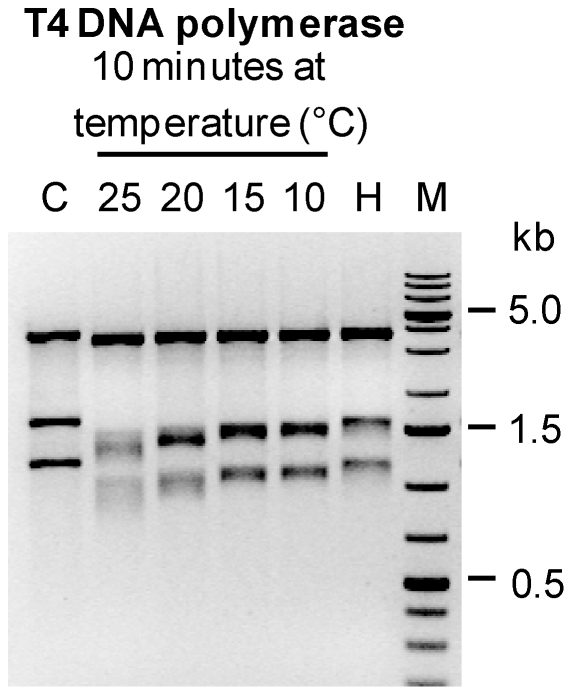
Quantification of T4 DNA polymerase exonuclease activity. *Sac*II/*Nde*I-digested plasmid DNA (3 fragments, lane C) was treated with T4 DNA polymerase for 10 minutes at 25°C, 20°C, 15°C and 10°C. The T4 DNA polymerase was then inactivated by incubation at 80°C for 5 min. The single-stranded ends generated by the 3′ to 5′ exonuclease activity T4 DNA polymerase were removed by using Mung Bean nuclease. The size of the resulting fragments was analyzed by agarose gel electrophoresis. As a control for the heat inactivation of T4 DNA polymerase, digested plasmid DNA was inactivated at 80°C for 5 minutes immediately after addition of T4 DNA polymerase (lane H).

Before testing QC cloning, we first quantified T4 polymerase exonuclease activity to determine conditions that would be suitable to make regions K1 and K2 single-stranded. As an assay to measure exonuclease activity, DNA fragments of a control plasmid digested with *Sac*II and *Nde*I (3 fragments of size 3.6, 1.6 and 1.1 kb) were treated with T4 DNA polymerase for 10 minutes at various temperatures (25°C, 20°C, 15°C and 10°C), generating single-stranded DNA at the ends of each fragment. The fragments were then incubated with Mung bean nuclease to remove the single-stranded extensions. The size of the fragments was then estimated by gel electrophoresis. All incubations resulted in a shift to a lower size, with stronger shifts obtained with higher temperatures (**Fig. 2**). 10 minutes incubation at 25°C resulted in digestion of approximately 100 to 300 nucleotides (visible as a smear). Considering that digestion must take place at both ends of each fragment, a single-stranded region of approximately 50 to 150 nucleotides must be present at each end of the linear fragments after T4 treatment. Therefore, incubation of 10 minutes at room temperature should be amply sufficient for QC cloning.

### QC cloning can be performed in one tube and one step

**Figure 3:**
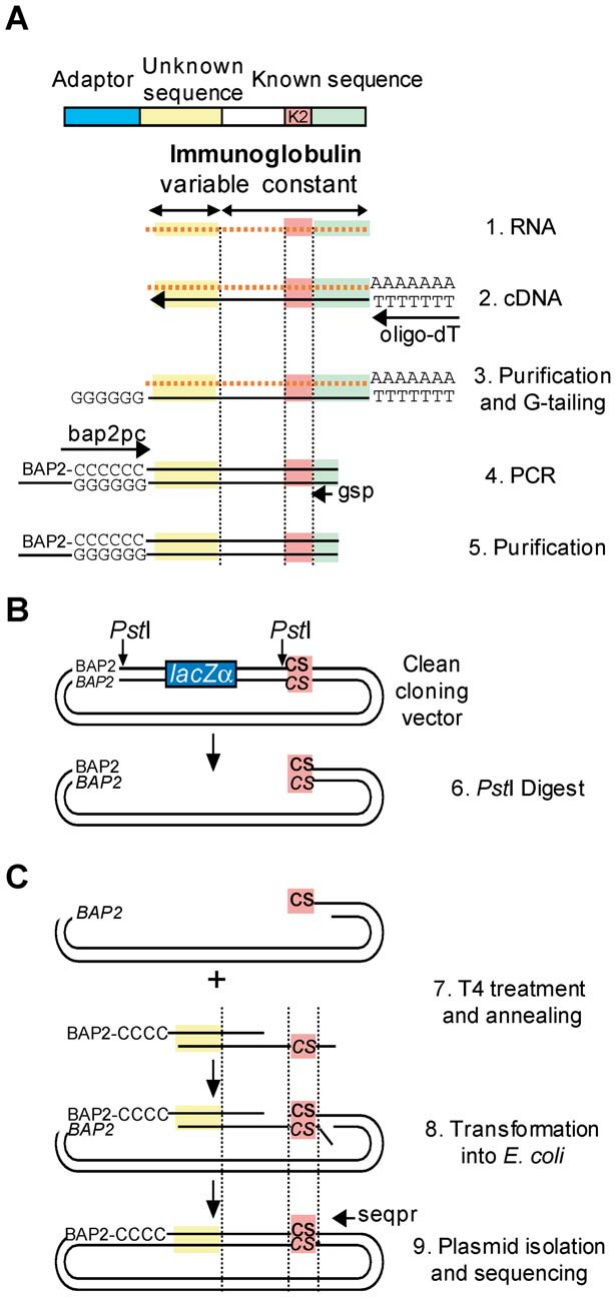
Strategy for amplification and QC cloning of immunoglobulin fragments. (**A**) Amplification of immunoglobulin fragments from non-Hodgkin lymphoma samples. Total RNA extracted from biopsy samples (1) is reverse-transcribed into first strand cDNA using an oligo dT primer (2). The cDNA is column-purified to remove remaining dNTPs, and G-tailed using terminal transferase and dGTP (3). (4) The G-tailed cDNA is used as a template for PCR amplification using a G-tail adaptor primer (bap2 pc) and an immunoglobulin constant region-specific primer (gsp). The PCR product is column-purified to remove the remaining dNTPs (5). (**B**) Preparation of vector for QC cloning. The cloning vector is linearized using the enzyme *Pst*I. (**C**) The column-purified PCR product and the linearized vector are mixed and treated with T4 DNA polymerase to generate single-stranded ends that are complementary between the vector and insert (7). The mixture is directly transformed into chemo-competent *E. coli* DH10B cells where the annealed ends of the vector and insert complex are repaired and ligated (8). (9) After cloning, the plasmid is purified and the insert sequenced using a vector specific primer (seqpr).

As target sequences for cloning, we amplified the variable region of immunoglobulins expressed in lymph node biopsies from non-Hodgkin lymphoma patients. The protocol we used for amplification is similar to a protocol described previously for performing 5′ RACE [12], but requires only one round of PCR (**Fig. 3A**). First-strand cDNA is made from total RNA using an oligo-dT primer and reverse transcriptase. A G-tail is then added at the 3′ end of first-strand cDNAs (corresponding to the 5′ end of the transcript) using terminal transferase. A PCR product containing the entire variable region (the unknown sequence) and part of the constant region (the known sequence) of the immunoglobulin is amplified by PCR using an adaptor primer (bap2 pc, consisting of a 19 nt adaptor sequence, bap2, followed by 14 cytosines) and a constant region-specific primer (gsp). The cloning vector contains the bap2 and CS (homologous to K1) sequences on either sides of a *lacZ*α gene fragment (used for blue-white selection). Digestion with *Pst*I excises the *lacZ*α gene fragment and produces vector ends that are accessible to T4 DNA polymerase for exonuclease digestion (**Fig. 3B, C**).

**Figure 4:**
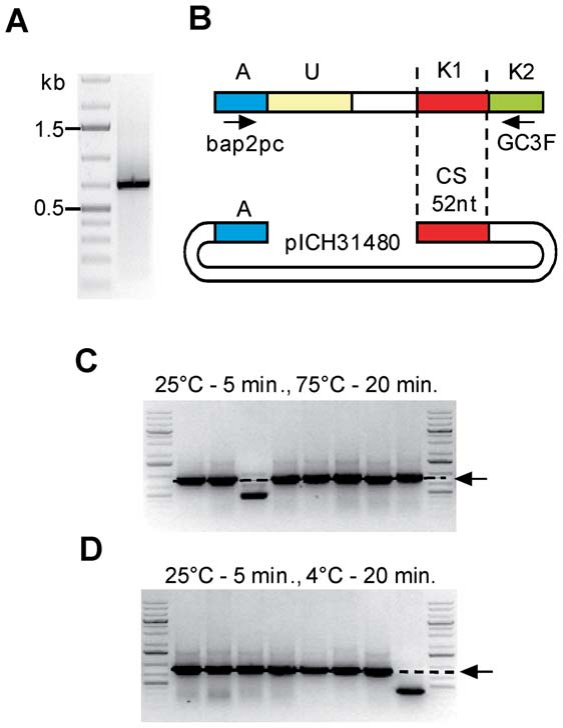
Test of QC cloning performed with or without heat inactivation. (**A**) PCR product amplified from G-tailed cDNA prepared from biopsy sample T019 using primers bap2 pc and GC3F. (**B**) Structure of the vector and of the PCR product. (**C**, **D**) The PCR product was cloned into pICH31480 using T4 DNA polymerase treatment for 5 minutes at 25°C (A, adaptor; U, unknown sequence; K, known sequence; CS, catching sequence), followed by heat inactivation 20 min at 75°C (**C**) or incubation at 4°C (**D**). Eight randomly chosen clones were analyzed by colony PCR using vector primers. The products amplified by colony PCR were separated on a 1% agarose gel supplemented with ethidium bromide and visualized under UV light. The expected insert size is indicated by an arrow.

A PCR fragment was amplified from non-Hodgkin lymphoma biopsy sample T019 using primers bap2 pc and GC3F (binds to the constant region of IgG immunoglobulins), column purified, and an aliquot of the purified product checked on an agarose gel (**Fig 4A**). To perform QC cloning, 2 µl of column-purified PCR product (5–50 ng), 1 µl of unpurified *Pst*I-digested cloning vector (pICH31480, 5–20 ng, the digested mix contains both the vector backbone and the *lacZ*α fragment), **2** µ**l** of 10x T4 ligase buffer, 0.5 µl T4 DNA polymerase (10 units) and 14.5 µl water were mixed in one tube and incubated for 5 min at 25°C. One mix was further incubated at 75°C for 20 min to inactivate the T4 DNA polymerase, while another was kept at 4°C for 20 minutes. The samples were directly transformed into 100 µl chemo-competent *E. coli* DH10B cells and 1/20 of the transformation was plated on selective medium.

Sixty seven white and no blue colonies were obtained for the experiment with heat inactivation, and 173 white and no blue colonies were obtained for the experiment without heat inactivation. Eight white colonies for each transformation were screened by colony PCR with vector primers. In both cases, seven out of eight clones contained an insert of the expected size (**Fig. 4C, D**). The shorter products were also specific, but contained 5′ truncated immunoglobulin fragments (see below). This first experiment tells us that the one-tube QC cloning protocol allows cloning of PCR products, and that the heat inactivation step is not required. Therefore, heat inactivation was omitted in the following experiments.

### Catching sequences of various lengths can be used for QC cloning

**Figure 5:**
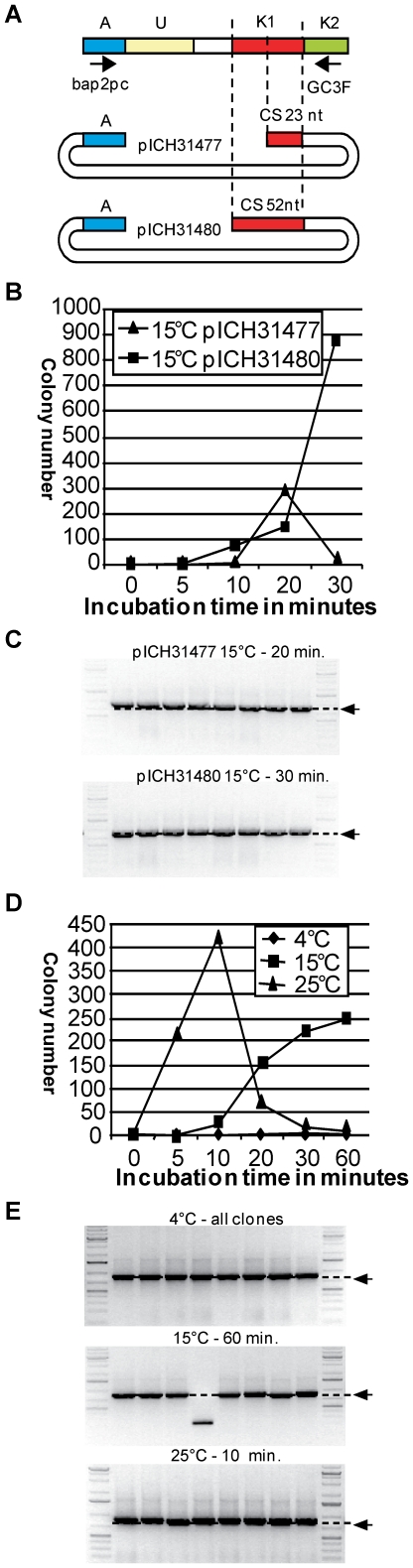
Test of CS length, reaction temperature and incubation time. (**A**) To analyze the influence of the catching sequence length on cloning efficiency, the T019 GC3F PCR product was cloned into pICH31477 (23 nucleotide CS) and pICH31480 (52 nucleotide CS). Cloning was performed using an incubation at 15°C for 0, 5, 10, 20 and 30 minutes (A, adaptor; U, unknown sequence; K, known sequence; CS, catching sequence) (**B**). (**C** and **E**) Eight randomly chosen clones from reactions with the incubation times that yielded the most clones were analyzed by colony PCR using vector primers. The PCR products were separated on a 1% agarose gel supplemented with ethidium bromide and visualized under UV light. The expected insert size is indicated by an arrow. (**D**) To determine the optimal incubation temperature and time, the T019 GC3F PCR product was cloned into pICH31480 (52 nucleotide CS) using incubation temperature of 4°C, 15°C and 25°C for 0, 5, 10, 20, 30 and 60 minutes.

To test the influence of the length of the CS on the efficiency of QC cloning, the T019 PCR product described above was cloned in two cloning vectors, pICH31477 with a CS of 23 nucleotides and pICH31480 with a CS of 52 nucleotides (**Fig. 5A**). QC cloning was carried out for 0, 5, 10, 20 and 30 minutes at 15°C. A temperature of 15°C rather than 25°C was selected for this experiment in order to reduce the speed of exonuclease digestion, and test whether such limiting conditions would also be able to make the entire 52 nt CS single-stranded (a total of 52+34 nt), as required for cloning. After exonuclease digestion, the samples were directly transformed into 100 µl chemo-competent *E. coli* DH10B cells, and 1/20 of the transformation was plated on selective medium. Maximum yields of 880 and 290 clones were obtained with 30 minutes incubation with vector pICH31480, and with 20 minutes incubation with vector pICH31477, respectively (**Fig. 5B**). Colony PCR performed on eight white colonies per transformation using vector primers (**Fig. 5C**) showed that all constructs obtained contained inserts of the expected size. Therefore, CSs of various lengths can be used for QC cloning. The 52 nucleotide CS requires a longer incubation time as compared to the shorter 23 nucleotide CS to produce the maximum number of white colonies.

### QC cloning can be carried out efficiently at room temperature

To find the optimal temperature and incubation time combination for QC cloning, the T019 GC3F PCR product was cloned in pICH31480 (**Fig. 5A**) using incubation times of 0, 5, 10, 20, 30 and 60 minutes at 4°C, 15°C and 25°C. The samples were then directly transformed into 100 µl *E. coli* DH10B and 1/20 of the mixture was plated on selective medium. The highest number of positive clones was obtained with an incubation of 10 min at 25°C (423 clones), whereas incubation for more than 20 minutes delivered only few clones (**Fig. 5D**). It is possible that production of too long single-stranded sequences may lead to extensive secondary structure in both the insert and vector, which may inhibit annealing of vector and insert. In addition, incubation at 25°C for a long time may also lead to complete digestion of the insert. A maximum of 249 clones were obtained after 60 minutes incubation at 15°C. Incubation at 4°C delivered only a background level of 0 to 3 clones at each time point, possibly resulting from T4 DNA polymerase exonuclease activity occurring during the pipetting steps that were not carried out on ice. Eight clones per experiment were analyzed by colony PCR and all except one (15°C, 60 minutes) were found to contain an insert of the expected size (**Fig. 5E**). Therefore, QC cloning can be set up on ice with little or no exonuclease activity occurring, and then carried out at 25°C for 10 minutes.

### Comparison of the specificity of blunt-end cloning, LIC and QC cloning

**Figure 6:**
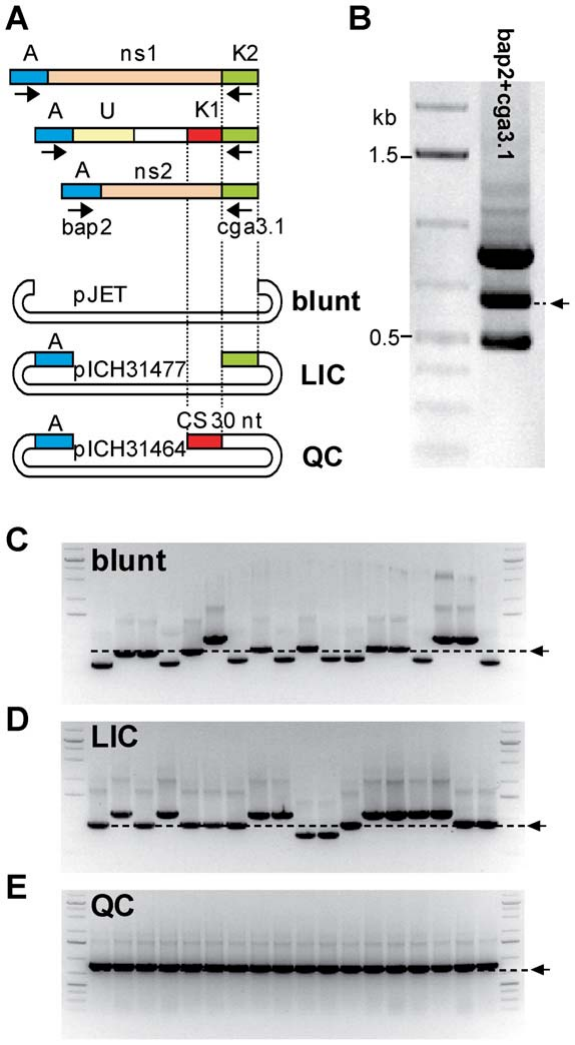
Comparison of blunt-end cloning, ligation-independent cloning and QC cloning using a mix of defined PCR products. (**A**) To compare efficiency of the three cloning methods, a mixture of three PCR products, two unspecific (ns1 and ns2) and one immunoglobulin fragment, was cloned (A, adaptor; U, unknown sequence; K, known sequence; ns, non-specific; CS, catching sequence). (**B**) PCR product mix amplified using primers bap2 pc and cga3.1. The arrow indicates the immunoglobulin fragment. (**C, D, E**) 12 randomly chosen clones for each cloning strategy were analyzed by colony PCR using vector primers. The immunoglobulin insert size is indicated by an arrow and a dashed line. (**C**) Unspecific blunt-end cloning into pJET1.2. (**D**) ligation-independent cloning into pICH31477 using a CS identical to cga3.1. (**E**) QC cloning into pICH31464.

To compare the specificity of QC cloning, LIC and blunt-end cloning, a mix containing three defined PCR products, including a specific product (an immunoglobulin fragment) and two artificially constructed non-specific products (ns1 or ns2, **Fig. 6A**), was used for cloning. For preparation of the mix, all three DNA fragments were amplified separately using primers bap2 and cga3.1, column-purified and mixed in equimolar amounts (**Fig. 6B**). The PCR product mix was then cloned into pJET1.2 (blunt-end cloning vector), pICH31477 (LIC vector) and pICH31464 (QC cloning vector). pJET cloning was carried out according to the manufacturer's protocol for 10 minutes at room temperature. To use comparable conditions, both LIC and QC cloning reactions were set up as described above and incubated in a PCR block for 5 minutes at 25°C. Both reactions were transformed directly into chemo-competent *E. coli* DH10B cells. For each cloning, more than 500 clones were obtained. 18 colonies per cloning reaction were analyzed by colony PCR using vector primers (**Fig. 6C**–**E**). With the blunt-end cloning vector pJET1.2, all three PCR products were cloned, however, with a preference for the shorter fragments (**Fig. 6C**). For the LIC vector pICH31477 all three PCR products were also cloned. However, in contrast to blunt-end cloning, LIC lead to cloning of a higher proportion of larger inserts (**Fig. 6D**). As expected, QC cloning resulted in cloning of the immunoglobulin fragment only (**Fig. 6E**).

### Comparison of the specificity of blunt-end cloning, LIC and QC cloning on immunoglobulin fragments of sample T044

**Figure 7:**
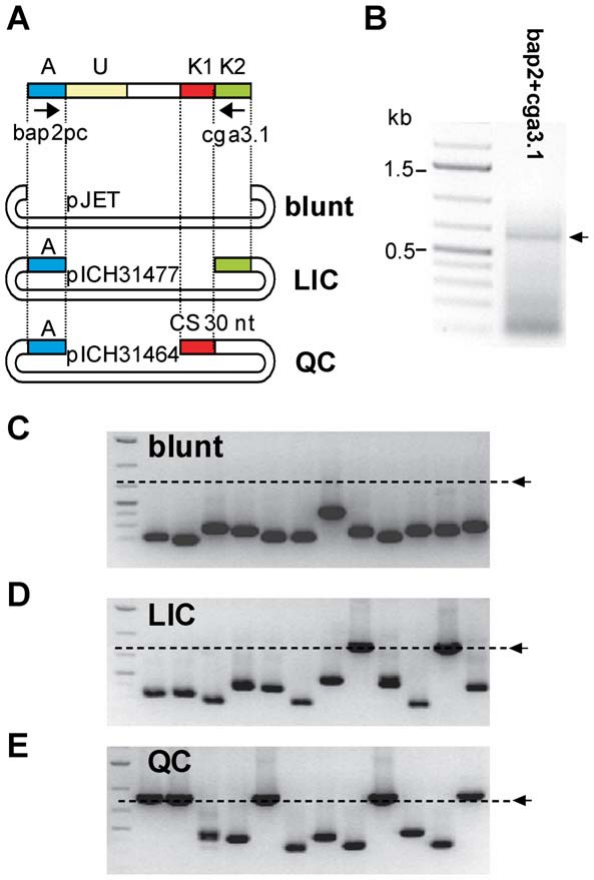
Comparison of blunt-end cloning, ligation-independent cloning and QC cloning on sample T044. (**A**) To compare the efficiency of the three cloning methods, a PCR product amplified from G-tailed cDNA prepared from sample T044 was cloned using blunt-end, LIC and QC cloning (A, adaptor; U, unknown sequence; K, known sequence; ns, non-specific; CS, catching sequence). (**B**) PCR product amplified from T044 G-tailed cDNA using primers bap2 pc and cga3.1. (**C, D, E**) 12 randomly picked clones for each cloning strategy were analyzed by colony PCR using vector primers. The size of the expected immunoglobulin fragment is indicated by an arrow and a dashed line.

The next experiment was performed to compare the specificity of QC cloning, LIC and blunt-end cloning for cloning of a PCR fragment amplified from a non-Hodgkin lymphoma biopsy sample (T044, amplified using primers bap2 pc and cga3.1, **Fig. 7B**). The PCR product was amplified with a relatively low Tm of 32°C to decrease the specificity of the amplified PCR product. The PCR product was then cloned into pICH31477 (LIC) and pICH31464 (QC cloning) and into pJET1.2 (blunt-end cloning). The 23 nucleotide sequence at the end of pICH31477 is identical with the sequence of primer cga3.1 (**Fig. 7A**). Blunt-end cloning of the PCR product into pJET1.2 resulted in a high number of clones, but colony PCR of 12 randomly chosen clones showed the presence of only inserts smaller than the expected size for full-length immunoglobulin fragments (**Fig. 7C**). LIC and QC cloning produced 500 and 246 clones, respectively. It is possible that a lower number of clones was obtained using QC cloning since only a fraction of the amplified products were specific and therefore cloneable. Colony PCR on clones obtained from both cloning experiments showed the presence of inserts of the size expected for full length products (**Fig. 7D, E**).

Inserts of 48 clones obtained with LIC and QC cloning were sequenced. For the LIC inserts, sequences of 45 clones were obtained, 26 (57.8%) of which contained immunoglobulin sequences. Twelve of these (26.7% of the total) contained full-length variable region sequences, while 19 (42.2%) contained non-specific sequences unrelated to immunoglobulins. For QC cloning, sequences of 47 clones were obtained, 100% of which corresponded to immunoglobulin sequences. Out of the 47 sequences, 30 (63.8%) contained full-length variable regions. These results showed the efficiency and specificity of the QC cloning method even when PCR leads to amplification of mixtures of specific and non-specific sequences.

### Use of QC cloning for cloning of Gamma, Mu, Kappa and Lambda immunoglobulin fragments

**Figure 8:**
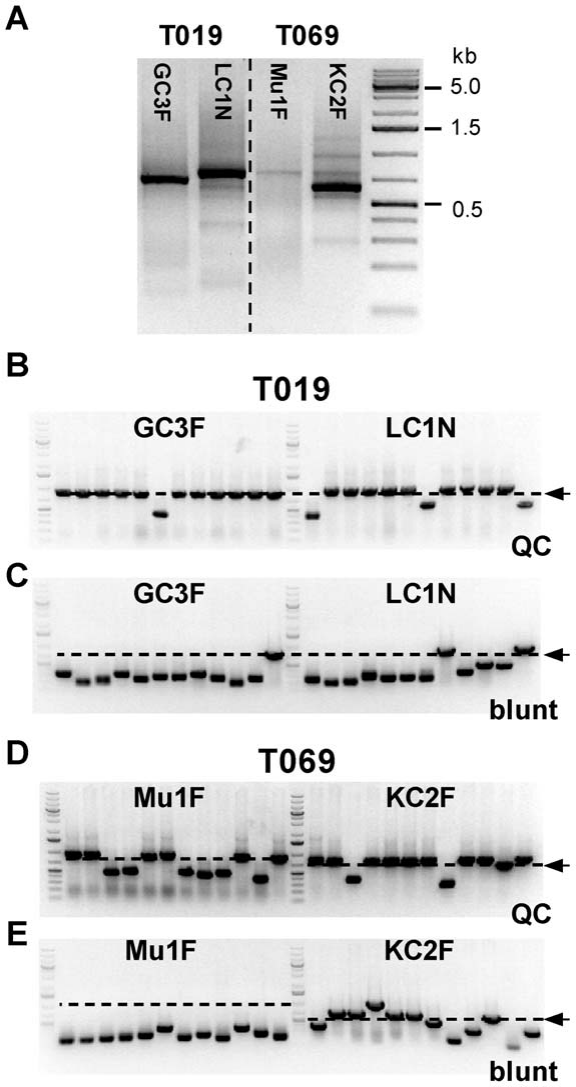
Cloning of Gamma, Mu, Kappa and Lambda immunoglobulin fragments by blunt-end and QC cloning. (**A**) PCR products amplified from G-tailed cDNA prepared from non-Hodgkin lymphoma biopsy sample T019 (isotype Gamma, Lambda) and T069 (isotype Mu, Kappa). Amplification was performed using primer bap2 pc and primers GC3F, LC1N, Mu1F and KC2F as indicated. The PCR products were cloned using QC cloning (**B and D**) or blunt-end cloning (**C and E**). 12 randomly chosen clones for each reaction were analyzed by colony PCR using vector primers. The expected size of full-length inserts is indicated by a dashed line and an arrow.

To test QC cloning with a variety of targets, we cloned the variable regions of the heavy and light chains of immunoglobulins expressed in two non-Hodgkin lymphoma biopsy samples: T109, which contains a tumor-associated immunoglobulin of the isotype IgG,L (heavy chain Gamma, light chain Lambda), and T069, with the isotype IgM,K (heavy chain Mu, light chain Kappa). The fragments containing the variable regions of the heavy and light chains were amplified using the adaptor primer bap2 pc (in all cases) and the constant primers GC3F, LC1N, Mu1F and KC2F specific for the immunoglobulin fragments of classes Gamma, Lambda, Mu and Kappa, respectively (**Fig. 8A**). Each of the PCR products were then cloned in corresponding QC cloning vectors (pICH31480 for the Gamma fragment and three other QC vectors with CSs of 49 or 50 nt specific for each immunoglobulin chain type). In parallel, the PCR products from all four amplifications were also cloned blunt-end into pJET1.2 as a control, to provide an overview of the composition of all amplified products, specific and non-specific.

In order to assess the quality of the cloning reactions, 12 randomly chosen clones from each cloning were analyzed by colony PCR using vector primers (**Fig. 8B**–**E**). For all four targets, a higher proportion of clones containing inserts of the expected size was obtained by QC cloning (6–11 out of 12) than by blunt-end cloning (0–5 out of 12). To analyze the cloned inserts in more detail, additional randomly chosen pJET1.2 and QC clones from each cloning reaction were picked and sequenced (**Table 1**). Out of 169 sequenced pJET1.2 clones, 27 (16%) contained the desired full-length immunoglobulin variable regions, 88 (52%) contained 5′ truncated immunoglobulin variable region fragments and 49 (29%) contained non-specific inserts (15 [9%] primer dimers, 34 [20%] sequences not corresponding to immunoglobulins) and 5 (3%) were empty vectors.

**Table 1:** Summary of sequencing data obtained for immunoglobulin fragments cloned by QC cloning or pJET blunt-end cloning.

	Sequenced/obtained	Full-length	Truncated	Primer dimer	Non-specific products	Empty vector
**QC cloning**						
T019 GC3F	32/30	24 (80%)	6 (20%)	-	-	-
T019 LC1N	24/24	12 (50%)	10 (42%)	-	1 (4%)	1 (4%)
T069 Mu1F	32/32	14 (44%)	18 (56%)	-	-	-
T069 KC2F	24/23	20 (87%)	3 (13%)	-	-	-
**pJET cloning**						
T019 GC3F	48/46	5 (11%)	11 (24%)	6 (13%)	23 (50%)	1 (2%)
T019 LC1N	48/37	4 (11%)	26 (70%)	2 (5%)	4 (11%)	1 (3%)
T069 Mu1F	48/40	-	25 (63%)	7 (18%)	7 (18%)	1 (3%)
T069 KC2F	48/46	18 (39%)	26 (57%)	-	-	2 (4%)
**Summary**						
pJET blunt- end cloning	192/169	27 (16%)	88 (52%)	15 (9%)	34 (20%)	5 (3%)
**QC cloning**	**112/109**	**70 (64%)**	**37 (34%)**	**-**	**1 (1%)**	**1 (1%)**

For QC cloning 107 (98%) out of 109 sequences obtained contained immunoglobulin sequences, 70 (64%) of which were full-length, and 37 (34%) 5′ truncated. Only one insert sequence (1%) did not correspond to an immunoglobulin sequence, but had partial homology to the CS, explaining its occurrence. One sequence corresponded to an empty vector (“religated” at the *Pst*I site). It should be noted here that the cloning experiments described here were carried out for testing the cloning procedure and its efficiency, and therefore, older biopsy material which contained partially fragmented mRNAs was used. When fresh biopsy material is used, more than 85% of the clones obtained with QC cloning contain full-length variable region fragments (2049 analyzed sequences have been cloned so far from 72 different PCR products; these sequences represent a total of 1755 full-length variable regions; data not shown). These results show both the specificity of QC cloning and the higher efficiency for cloning larger inserts as compared to other cloning strategies such as blunt-end cloning. PCR products derived from 5′ truncated transcripts can still be cloned with QC cloning, since these are nevertheless specific (they contain the constant region corresponding to the CS).

### QC cloning can be performed using Klenow polymerase

**Figure 9:**
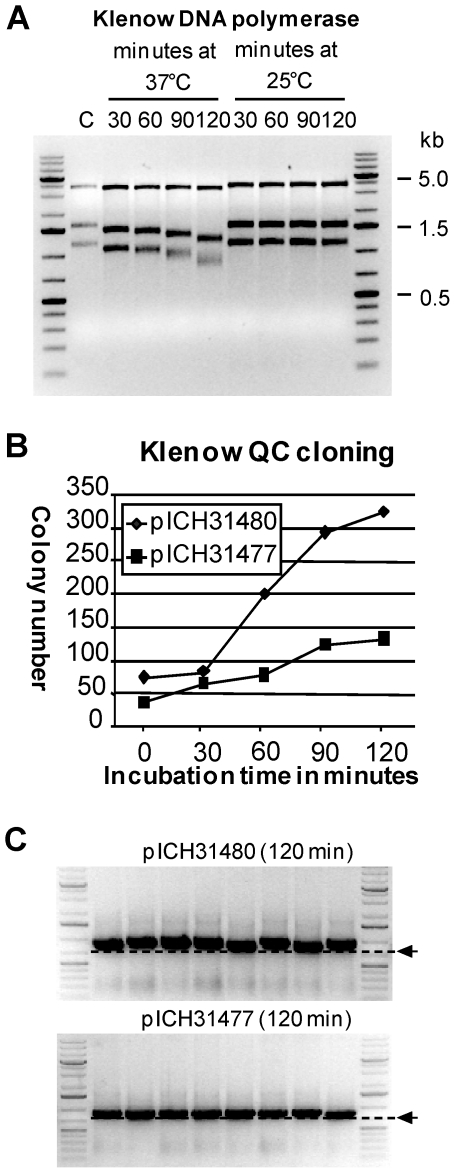
Test of QC cloning using Klenow DNA polymerase. (A) Test of Klenow exonuclease activity determined using the same assay used for T4 DNA polymerase. (B) To test QC cloning using Klenow DNA polymerase, the PCR product T019 GC3F was cloned into pICH31477 (23 nucleotide catching sequence) and pICH31480 (52 nucleotide catching sequence). Incubation was performed at 37°C for 0, 30, 60, 90, and 120 minutes. (**C**) Eight randomly chosen clones from 120 min time points were analyzed by colony PCR using vector primers. The size of the expected full-length fragment is indicated by an arrow.

Klenow polymerase is known to have a weaker exonuclease activity than T4 DNA polymerase. Therefore, it is expected that Klenow polymerase could be used as an alternative to T4 DNA polymerase in case more controllable exonuclease digestion conditions are needed. The T4 DNA polymerase exonuclease activity assay described above was used to quantify Klenow polymerase exonuclease activity under various conditions. *Sac*II/*Nde*I-digested plasmid DNA was incubated 30, 60, 90, and 120 minutes in the presence of Klenow polymerase at 25°C and 37°C. We found that no significant exonuclease activity could be detected at room temperature (25°C) (**Fig. 9A**). In contrast, single-stranded regions are formed at 37°C with incubations ranging from 30 to 120 minutes. This observation shows that almost no exonuclease activity will occur while pipetting (standard laboratory conditions of 20 to 25°C), while exonucleolytic activity will be obtained when the reaction is incubated at 37°C.

To determine the optimal incubation time and CS length for Klenow QC cloning, immunoglobulin fragments of sample T019 amplified using primer bap2 pc and GC3F were cloned into pICH31477 (23 nt CS) and pICH31480 (52 nt CS) (**Fig. 5A**). Klenow QC cloning was carried out for 0, 30, 60, 90, and 120 minutes at 37°C. The samples were directly transformed into 100 µl *E. coli* DH10B and the entire transformation was plated on selective medium. The number of white colonies increased with incubation time (**Fig. 9B**). All colonies tested by colony PCR contained an insert of the expected size for both cloning vectors (**Fig. 9C**). QC cloning can therefore be performed using Klenow polymerase, but requires a significantly longer incubation as compared to T4 DNA polymerase.

## Discussion

Ligation-independent cloning was first described two decades ago [8] and is now increasingly used for a number of applications [13], [14], [15], [16], [17], [18], [19]. LIC offers many advantages: (1) it does not require cleavage of insert DNA with restriction enzymes, and therefore can be used to clone libraries of unknown sequences, (2) it is highly efficient, in part because empty vector cannot religate without insert, but also because annealing of long single-stranded DNA ends (of more than 12 nucleotides) allows direct transformation in *E.coli* cells without the need for an *in vitro* ligation step [11], [20]. The initial LIC protocol required the use of specific sequences at the ends of the vector and insert that lacked a specific nucleotide, and also required incubation of vector an insert separately with T4 DNA polymerase in the presence of the specific deoxynucleotide missing in the sequence; such protocol was designed to restrict the length of DNA ends made single stranded [8], [20]. Since the first publication, a number of improvements have been made. Yang *et al*. [10] showed that T4 DNA polymerase treatment can be performed without the addition of any nucleotide in the reaction mix, thereby removing the requirement to use specific sequences lacking one of the 4 nucleotides. Since the single-stranded extension might be longer than the 12–15 nucleotides of the overlap region, the annealed vector-insert complex was treated with T4 DNA polymerase using all 4 deoxynucleotides to fill the single-stranded gaps. In a separate work, Li and Evans [11] also performed an exonuclease treatment, this time using Exonuclease III rather than T4 DNA polymerase, and, as in the work of Yang et al, treatment was performed in the absence of any nucleotide. However, they did not fill the gaps left at the junction sites, and showed that bacterial cells were able to both trim away non-hybridized overhang sequences and fill and repair the gaps. Later, Li and Elledge [21] showed that a strategy called sequence and ligation independent cloning (SLIC), that is performed with T4 DNA polymerase on DNA fragments with overlapping ends consisting of any native sequence could be used to assemble up to 9 PCR-amplified fragments into a vector.

We have further optimized parameters of this cloning method. First, and unlike other described LIC protocols, the entire procedure is performed in one tube and in one step: PCR product and linearized vector are pipetted into one tube together with T4 DNA polymerase, incubated 10 minutes at 25°C and directly transformed into competent cells. This streamlined protocol is nevertheless still very efficient, and we found that adding an extra step to inactivate T4 DNA polymerase before transformation did not increase cloning efficiency. Furthermore, we tested whether the *E. coli* large Klenow fragment could be used for exonuclease digestion instead of T4 DNA polymerase. As expected, we found that Klenow could indeed be used for ligation-independent cloning. Interestingly, the exonuclease activity of Klenow is too low to work at room temperature, and incubation needs to be performed at 37°C. This can be useful, since it makes it easier to set up many cloning mixes without having to worry that exonuclease starts working before finishing pipetting for all samples. However, for most applications, the use of T4 DNA polymerase is still preferable as cloning can be done faster.

More importantly, we have shown here that use of a vector designed to not have any homology with one of the primers used for amplification of the insert can be used to develop a protocol, called QC cloning, that allows cloning of specific products only. Interestingly, this was achieved without reducing the cloning efficiency that is normally obtained with standard LIC vectors. We have tested this protocol on an artificial mix of PCR products containing one third of specific products and two third of non-specific products and found that only specific products were cloned. We have also tested this cloning procedure on lymph node biopsy samples of non-Hodgkin lymphoma patients to identify the variable regions of immunoglobulins, and found that here as well, virtually only specific sequences were cloned. This feature makes it more efficient to identify the tumor-associated idiotype (the variable region of the specific immunoglobulins present on tumor cells), since, because all white colonies contain a specific insert, screening for colonies before sequencing is no longer necessary. Furthermore, the ability to clone exclusively specific products removes the need for nested PCRs that are normally required to increase the ratio of specific to non-specific products. This in turn reduces the chance for biasing the population of amplified sequences, which might happen if for example a polymorphism is present in the target sequence of one of the nested primers.

We have also performed a more general comparison of the efficiency of three different cloning methods: blunt-end cloning, ligation-independent cloning and QC cloning. Interestingly, we found that standard blunt-end cloning leads to preferential cloning of small products, while both LIC and QC cloning are able to clone larger products more efficiently. This can be explained by the fact that with ligation-independent cloning, DNA fragments are digested at both ends by T4 DNA polymerase. At some point, some of the small fragments may become completely digested. Another explanation may be that with blunt-end cloning, once one end of the vector has been ligated to an insert, the ends of the resulting linear fragment are more likely to religate for smaller inserts that for large ones since the ends are less distant from each other. For ligation-independent and QC cloning, the two homologous regions at, or near, the ends of the vector and insert allow efficient annealing of the ends, and may therefore provide conditions less limiting on insert size for annealing of both ends of the vector-insert complex. In addition, with LIC and QC cloning, vector-insert complexes that may be annealed at only one end can easily anneal at the other end later after transformation in *E.coli*, provided that the two ends exhibit a region of homology. In contrast, with blunt-end cloning, linear fragment that consists of a vector molecule ligated with an insert at one end only cannot be religated in *E.coli*. Whatever mechanism is involved, this feature of ligation-independent cloning methods is very useful as it allows to increase the ratio of clones obtained that contain full-size inserts. For example, in case of amplification of the variable region of antibodies, products containing a complete variable region have a size of around 700 nt (depending of course on where primers are located in the constant region). However, smaller products can be obtained that contain 5′ truncated variable region fragments. These fragments can be formed either by reverse transcription of 5′ truncated transcripts, or when reverse transcription fails to proceed through the entire length of the transcript (for example because of RNA secondary structure). These incomplete first strand cDNAs will be tailed by terminal transferase as efficiently as full length cDNAs, and the PCR products derived from them will be cloneable, whether using blunt-end cloning or ligation-independent cloning. Fortunately, the push toward cloning of larger sequences that is observed with both ligation-independent cloning methods allows cloning the full size fragments more efficiently. Our experimental studies using biopsy samples showed this beneficial aspect of both ligation-independent cloning methods. It should be noted that in this study the biopsy material that was used had been stored for several years at −80°C, leading to a high amount of partially degraded or fragmented transcripts. However, even under these suboptimal conditions, more than 60% of the cloned fragments were full length. If fresh biopsy material is used, the amount of cloned full-length variable regions is well above 85% (data not shown).

We have investigated the length of the catching sequence and found that long CSs of 50 nucleotides can be used and even allow more efficient cloning. This result was not necessarily expected since with a 50 nucleotide long CS, a region of >70 nucleotides (50 nucleotides of the CS plus the primer region plus any sequence present between these two sequences) has to be made single stranded on one side of the insert. Not surprisingly, this requires using a slightly longer incubation time for optimal efficiency than when smaller catching sequences are used. Although using small 20 nucleotide long catching sequences works well under normal conditions, being able to use 50 nucleotides is advantageous since one can never be sure that polymorphisms are not present in the region of the insert DNA homologous to the catching sequence. For example, when antibody variable regions are cloned from patient biopsies, a catching sequence from the constant region is used. However, even the constant region of antibodies contains polymorphisms that vary between different patients, and even a database search will not be able to identify all possible polymorphisms. A polymorphism of one nucleotide in a sequence homologous to a 12 nucleotide catching sequence may prevent cloning of a product containing such sequence. In contrast, using a 50 nucleotides CS will allow any homologous sequence to be cloned, since a stretch of 12 conserved nucleotides will always be available. Long catching sequences might also be useful for other applications of QC cloning; for example, QC cloning might be used for cloning sequences from gene families. In that case, use of longer catching sequences will improve the chance of cloning homologues.

**Figure 10:**
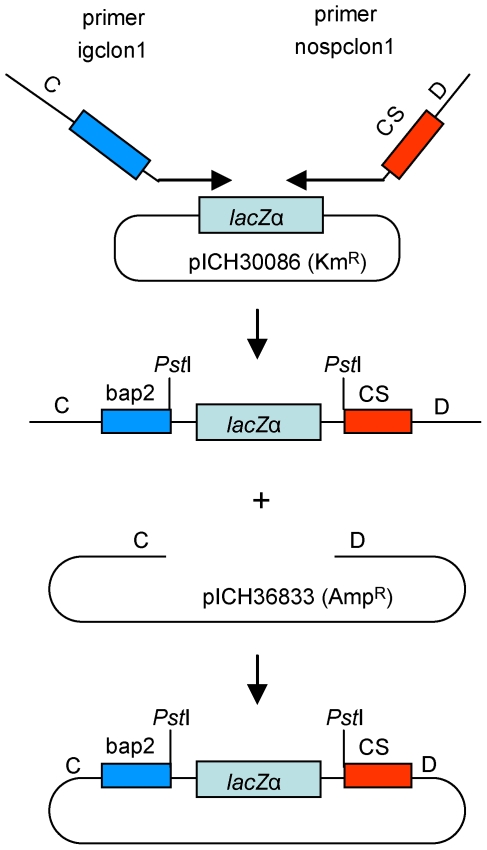
Construction of QC cloning vectors. QC cloning vectors can be prepared by amplification of a DNA fragment containing a visible selectable marker (a *lacZ*α fragment was used here) with two primers with 5′ extensions containing the bap2 sequence (blue box) and the catching sequence (CS, red box). The primers also contain extensions C and D with homology with any cloning vector of choice. The PCR product is cloned by ligation-independent cloning in a linearized vector (here pICH36833) with DNA ends homologous to sequences C and D. DNA from blue colonies are sequenced to make sure that no mutations are present in the sequence of bap2 and the CS.

One requirement for QC cloning is the construction of a specific vector for each new target. Such requirement is not difficult to satisfy since QC cloning vectors are easy to make, for example using ligation-independent cloning (any other alternative cloning technique, such as described in Engler *et al*. [22], would also work). For construction using ligation-independent cloning, only one new primer needs to be designed for each new vector (for example, nospclon1 shown in **Figure 10**), as the second primer corresponding to the adaptor sequence (primer igclon1) will be the same for all vectors. The new primer has to contain a 5′ sequence with homology to the vector backbone chosen for making the QC cloning vector (sequence D), then the CS, followed by a restriction site (*Pst*I in the example given) and finally by a sequence homologous to a fragment containing a *lacZ*α marker or any other favored marker or counter-selectable cassette. The PCR product is amplified and cloned in one tube and one step into a linearized vector backbone. To use the new vector, compatible primers for the amplification of the target sequence have to be designed. The CS does not have to be positioned exactly immediately after the primer used for amplification of the target sequence. For example, we have tested vectors in which the primer used for the amplification and the CS were separated by 10, 20 or even 30 nucleotides, and QC cloning did still work. This is useful since the same vector can be used for cloning PCR products amplified with different primers, as long as these primers are located not too far from the sequence homologous to the CS.

As an application, the QC cloning approach is currently used in the manufacturing process of personalized vaccines against non-Hodgkin lymphoma. QC cloning is used to identify the unique immunoglobulin variable regions present on the surface of the tumor B-cell lines. The variable regions are then subcloned to make chimeric full length antibody constructs, which are used for production of the recombinant immunoglobulins by transient expression in *Nicotiana benthamiana*. The recombinant immunoglobulin is then used as an antigen to elicit an immune response against the tumor cells [23].

The quick and clean cloning method that is presented here can also be used to clone and identify the flanking sequence of DNA elements such as transposons or T-DNA insertions. In such cases, an amplification procedure suitable for DNA templates has to be used, but the same QC cloning principle as described here for PCR products containing antibody variable sequences can be applied. Finally, it should be noted that the QC cloning procedure is not limited to the cloning of unknown flanking sequences, but can also be used for cloning of any other PCR product. For example, QC cloning may be useful for cloning and identification of gene family members; the use of QC cloning would be advantageous since the use of degenerate primers for amplification of several family members may result in mixtures of specific and non-specific products. QC cloning vectors can then be designed to catch a specific population from the pool of amplified sequences.

## Methods

### Ethics statement

Human non-Hodgkin lymphoma biopsy samples kindly provided by Maurizio Bendandi, Laboratory of Immunotherapy of the Center for Applied Medical Research (University of Navarra, Spain) were used as the starting material for cloning the immunoglobulin variable regions. Research use of the provided biopsy material has been approved by the Investigational Review Committee of University of Navarra Hospital (Comite' de Investigacion Clinica de la Clinica Universitaria de Navarra) with written informed consent being on file. The clinical samples have been analyzed anonymously.

### Construction of QC cloning vectors

For the construction of pICH31480, the *lacZ*α fragment was amplified by PCR from pUC19 with the primers Igclon1 (5′-GGA GGG TTG AAG ACT T **GTC CAG AGC CGT CCA GCA A** *CTG CAG* GCA GCT GGC ACG ACA GGT TTC-3′) and Igclon4 (5′-GAT CCT AGA TGT GGA AGA CTT TAC **CAC GAC ACC GTC ACC GGT TCG GGG AAG TAG TCC TTG ACC AGG CAG CCC AGG G** *CTG CAG* CGC GCG TTT CGG TGA TGA-3′). The 3′-part of both primers (underlined) is specific for the *lacZ*α cassette. This sequence is preceded by a *Pst*I recognition site (italics). The middle part (bold dotted line) contains the homologous sequences for QC cloning, bap2 in Igclon1 and the CS in Igclon4, a 52 bp region of the first exon of the IgG constant region. The 5′-end of each primer is homologous to the ends of a linearized vector used for cloning, in this case pICH29965 (carbenicillin resistance, pUC19 origin, no *lacZ*α). The *lacZ*α PCR product and the *Bpi*I-digested (Fermentas, St. Leon-Rot, Germany) vector pICH29965 were column purified (NucleoSpin Extract II, Macherey-Nagel, Düren, Germany). To perform the QC cloning 2 µl PCR product, 1 µl *Bpi*I-digested vector, 2 µl 10x T4 DNA polymerase buffer, 0.5 µl T4 DNA polymerase (New England Biolabs, Ipswich MA, USA; 3 units/µl) and 14.5 µl water were mixed and incubated for 5 minutes at room temperature. The mix was transformed in chemically competent *E. coli* DH10B cells and plated on media containing 40 µg/ml X-Gal (Sigma-Aldrich, Munich, Germany) and 250 µg/ml carbenicillin (Duchefa, Haarlem, The Netherlands). Positive clones were detected using blue/white selection, with blue colonies containing the desired vector construct. The region containing the bap2 and the CS sequence was confirmed by sequencing before use.

All other QC cloning vectors were prepared following a similar protocol, but primer Igclon4 was replaced by other primers with different CSs.

### Processing of non-Hodgkin lymphoma samples

The protocol from Osterroth *et al.* [12] was modified. RNA was isolated from non-Hodgkin lymphoma single-cell suspensions using the RNeasy kit (Qiagen, Hilden, Germany). 0.5–1.0 µg RNA was reverse transcribed into cDNA using the SuperScript III reverse transcriptase kit and Oligo dT20 (Invitrogen, Carlsbad, California, US). The resulting cDNA was not treated with RNaseH, but column-purified (MN Extract II kit, Macherey-Nagel) prior G-tailing using terminal transferase (New England Biolabs). The amplification of antibody fragments from G-tailed first-strand cDNA was carried out using a G-tail-specific primer (bap2 pc: GTC CAG AGC CGT CCA GCA A CC CCC CCC CCC CCC) and immunoglobulin-specific primers derived from the respective constant region (for IgM, Mu1F: CTT GGA AGG CAG CAG CAC CTG; for IgG, GC3F: GGT GTG CAC GCC GCT GGT CAG and cga3.1: CAC GAC ACC GTC ACC GGT TC; for IgK, KC2F: GTG ACA CTC TCC TGG GAG TTA C; and for IgL, LC1N: CGG TGC TCC CTT CAT GCG TGA C). The variable region was amplified using KOD Hot Start DNA polymerase (Merck, Darmstadt, Germany) with the following parameters: 95°C for 2 min, followed by 40 to 45 cycles of 95°C for 20 sec, 58°C for 10 sec, and 70°C for 20 sec, followed by a final incubation at 70°C for 20 sec. The PCR products were analyzed by agarose gel electrophoresis and column-purified (MN Extract II kit, Macherey-Nagel) to remove primers and the remaining dNTPs prior cloning.

### QC cloning

Cloning vectors were isolated from 1 ml stationary phase *E. coli* DH10B using the Nucleospin Plasmid QuickPure kit (Macherey-Nagel). 3 µl of the purified vector DNA (50–200 ng/µl) were digested in a 30 µl reaction with *Pst*I (New England Biolabs) for 2 h at 37°C, heat inactivated for 20 minutes at 80°C and analyzed by agarose gel electrophoresis. To perform cloning, 2 µl column-purified PCR product (5–50 ng/µl), 1 µl unpurified *Pst*I-digested cloning vector (5–20 ng/µl), 2 µl 10x T4 ligase buffer (New England Biolabs) or NEB buffer 2 (both buffers were tested and are suitable for QC cloning), 0.5 µl T4 DNA polymerase (New England Biolabs, 3 units/µl) and 14.5 µl water were mixed and incubated in a PCR block for 5–60 min at 4°C, 15°C or 25°C. Reactions were chilled on ice for 1 minute and directly transformed into 100 µl of chemo-competent *E. coli* DH10B cells. Clones were selected on LB agar plates supplemented with 40 µg/ml X-Gal (Sigma-Aldrich) and 250 µg/ml carbenicillin (Duchefa). Blue-white selection was used to identify white clones containing inserts, while blue clones contained undigested cloning vector. To analyze the cloned sequences, randomly chosen clones were tested by colony PCR using vector primers. Additionally, clones were sequenced using a vector primer.

### Generation of PCR products for comparison of cloning methods

To compare QC cloning, LIC and blunt-end cloning, a mix containing three defined PCR products, a specific immunoglobulin fragment and two artificially constructed non-specific products, was generated. The template for each PCR product was first cloned blunt-end in a standard vector which harbors a spectinomycin resistance gene. Each cloned template was flanked by bap2 and cga3.1 sequences, which were then used as primer binding sites for amplification from the plasmid template. The PCR products were column-purified and mixed in equimolar amounts.

### Blunt-end cloning into pJET1.2

Blunt-end cloning of PCR fragments was carried out using the CloneJET PCR Cloning Kit (Fermentas, St. Leon-Rot, Germany). Reactions were chilled on ice for 1 minute and transformed into 100 µl of chemo-competent *E. coli* DH10B cells. Clones were selected on LB agar plates supplemented with 250 µg/ml carbenicillin (Duchefa). To analyze cloning efficiency, randomly chosen clones were tested by colony PCR and sequencing.

### Colony PCR

Colony PCR was carried out using Hot Start *Taq* polymerase (Fermentas). Vector primers kanseqf (TGG AAA AAC GCC AGC AAC GC) and kanseqr (TGT CTC ATG AGC GGA TAC AT) were used for QC cloning vectors and pJET1.2 forward and reverse (see manufacturer's protocol) for pJET1.2 clones. PCR fragments were analyzed by agarose gel electrophoresis.

### Sequencing

Clones were sent to Eurofins MWG Operon (Martinsried, Germany) for plasmid isolation and sequencing. Primers kanseqf and pJET1.2 reverse were used for sequencing the insert of QC cloning vectors and pJET1.2, respectively.

### Bioinformatic analysis

Vector insert sequences were analyzed using BLAST [24]. Additionally, immunoglobulin variable region sequences were analyzed using IMGT/V-Quest [25].

### *In vitro* assay for quantification of T4 and Klenow DNA polymerase exonuclease activity

To quantify exonuclease activity, linear DNA was incubated with T4 or Klenow DNA polymerase. The DNA used was a *Sac*II/*Nde*I-digested plasmid (pICH10990), and consists of three fragments of 3595, 1563 and 1158 bp. 2.5 µl digested vector (∼150 ng) was treated with 1.5 units T4 DNA polymerase (New England Biolabs) in NEB buffer 2 for 10 minutes at 25°C, 20°C, 15°C and 10°C or 2.5 units Klenow polymerase (New England Biolabs) in NEB buffer 2 for 30, 60, 90 and 120 minutes at 25°C and 37°C. The incubation was followed by inactivation of the polymerase at 80°C for 5 minutes. Then, Mung Bean nuclease buffer and 10 units of Mung Bean nuclease (New England Biolabs) were added to the reaction mix. The reaction was incubated for 20 minutes at 25°C to remove single-stranded DNA overhangs. The treated DNA was then analyzed by agarose gel electrophoresis.

### Klenow QC cloning of immunoglobulin fragments

To perform Klenow QC cloning, 2 µl column-purified PCR product (5–50 ng/µl), 1 µl unpurified *Pst*I-digested cloning vector (5–20 ng/µl), 2 µl 10x NEB buffer 2, 2 µl Klenow polymerase (New England Biolabs, 5 units/µl) and 13 µl water were mixed and incubated in a PCR thermo cycler for 30–120 min at 37°C. Reactions were chilled on ice for 1 minute and directly transformed into 100 µl of chemo-competent *E. coli* DH10B cells.
